# Generating evidence and understanding the treatment of osteoarthritis in Brazil: a study through Delphi methodology

**DOI:** 10.6061/clinics/2019/e722

**Published:** 2019-05-27

**Authors:** Ibsen Bellini Coimbra, Pérola Grinberg Plapler, Gustavo Constantino de Campos

**Affiliations:** I Disciplina de Reumatologia, Departamento de Clinica Medica, Faculdade de Ciencias Medicas, Universidade Estadual de Campinas, Campinas, SP, BR.; II Instituto de Ortopedia e Traumatologia, Departamento de Fisiologia, Faculdade de Medicina (FMUSP), Universidade de Sao Paulo, Sao Paulo, SP, BR.; III Departamento de Ortopedia, Faculdade de Ciencias Medicas, Universidade Estadual de Campinas, Campinas, SP, BR.

**Keywords:** Osteoarthritis, Osteoarthrosis, Surgical Treatment, Drug Therapy, Glucosamine, Chondroitin

## Abstract

**OBJECTIVES:**

This study aimed to provide evidence for understanding how to treat osteoarthritis (OA) in our country. Therefore, it was necessary to match information and investigations related to the treatment of the disease from the three main types of specialists involved: physiatrists, orthopedists and rheumatologists.

**METHODS:**

The authors acted as a scientific advisory committee. From the initial discussions, a structured questionnaire was developed for use with a group of specialists on OA using the Delphi technique. The questionnaire was sent to 21 experts appointed by the authors, and the results obtained were critically analyzed and validated.

**RESULTS:**

The prevalence of OA was 33% in Brazil, corresponding to one-third of the individuals in the reference population, which included individuals over 25 years of age. Another significant finding was that most patients did not receive any form of treatment in the early stages of OA.

**CONCLUSION:**

The committee pointed to the need for early intervention and that the available medicinal resources can fulfil this important role, as is the case with SYSADOA treatments. Glucosamine-based medicinal products with or without chondroitin could also fulfill this need for early treatment. The other generated evidence and included investigations were then grouped together and are the subject of this publication.

## INTRODUCTION

Osteoarthritis (OA) or osteoarthrosis is a low-grade, progressive inflammatory disease that may entail joint degeneration (1) that affects all joint structures, notably the cartilage of synovial joints. OA evolves into joint insufficiency, characterized by changes in articular cartilage, with fibrillation and fissure zones, microfractures, cysts, subchondral sclerosis and formation of osteophytes on joint edges. These changes lead to chronic pain and functional constraints of the affected joints, in addition to the physical and psychological sequelae that often manifest in individuals with OA, causing greatly reduced quality of life.

OA is the most common joint disease (2,3) and affects mainly the knees, hips, hands and feet. In the USA, it is estimated that 36.4% of individuals over 60 years old have OA in their knees (4). This disease of great medical and economic importance is responsible for the inability to perform labor in approximately 15% of the adult population of the world. In Brazil, OA is currently the third most common disease of those who are insured for Social Security and receiving disease aid, corresponding to 65% of the cause of disability (5). The data are more relevant if we consider that the population of individuals older than 60 years, which was 19 million in 2012, will increase to more than 64 million in 2050 according to the Brazilian Institute of Geography and Statistics (IBGE) (6). These are extremely relevant data, considering the inability to work, reduced quality of life and costs to the health system generated by OA, including treating the disease and early retirement due to functional disability (7).

Until a few decades ago, the treatment of OA was limited to the use of simple analgesics, anti-inflammatory medications, physical measurements, steroid injections and, in refractory cases, surgical treatment. Understanding the pathophysiology of OA, that the pathogenesis is not purely mechanical and/or related only to aging, as well as the inflammatory pathways involved, has led to the clinical application of several novel treatments (8). These new treatments have significantly altered the clinical and therapeutic approach to OA.

This study aims to update our understanding of the disease in our country, to establish a general overview of OA, to suggest epidemiological indicators and to identify forms of treatment undertaken in Brazil. For this reason, a scientific advisory committee (SAC) was convened, consisting of specialists in the three areas directly involved in the detection, diagnosis and treatment of OA, namely, physiatry, orthopedics and rheumatology. These experts, in turn, included a total of 21 doctors in the most important regions of the country with remarkable knowledge about OA and composed the Delphi panel, from which the data presented here were obtained.

## MATERIALS AND METHOD

Initial discussions of the SAC led to the elaboration of a data collection form that, to fulfill the main objective, was used for the following goals: a) to capture epidemiological indicators of OA and b) to identify the use of different forms of treatment according to the affected joint and radiological of Kellgren and Lawrence classification. The questionnaire was then sent to the experts, including 8 physiatrists, 7 orthopedic surgeons and 6 rheumatologists.

### Delphi technique

This technique originated in 1966 (9) and is currently, to a large extent, used and intended for the deduction and refinement of opinions of a group of experts or specially educated individuals, with the objective of achieving a consensus of opinion by means of questions, feedback and review of responses.

The technique is defined as a “systematized method of information judgement” and is used to obtain expert consensus of a given theme through jointly phased or cycle-based validation. The Delphi technique is based on the structured use of the knowledge, experience and creativity of a panel of specialists, assuming that collective judgment, when organized, is better than the opinion of a single individual or of groups devoid of specific knowledge (9).

### Obtaining consensus

More than 28,000 data items were raised in the first wave of questionnaires collected. As a reflection of the homogeneity of the chosen panel, slightly more than 1% of the data points were classified as revisions (considered “outliers”). Of these, almost half have not been revised because they were not outliers when considering the averages in each subgroup (different specialties), signaling a typical difference in the subgroup and not a real outlier. In such cases, the global data were disregarded to demonstrate differences by specialty.

In the few remaining cases, one group was reviewed and another, due to a panel member not being able to review, was treated as “missing data” and handled with “pairwise” deletion, leading to the consensus (understood as the average near the median) presented below.

## RESULTS

### Epidemiology

**Figure 1:**
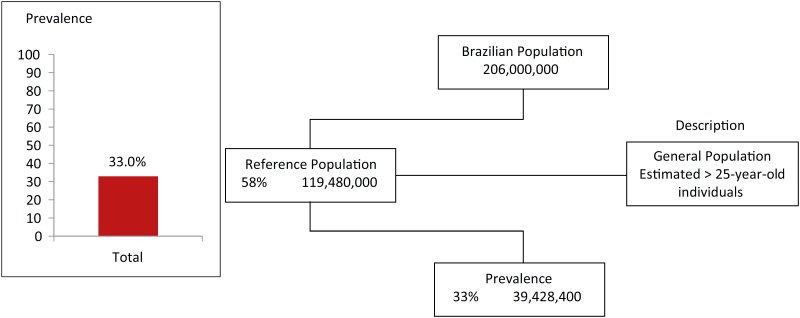
Prevalence of osteoarthritis in Brazil.

OA had a prevalence of 33% in Brazil. That is, one-third of the individuals in the reference population (individuals over 25 years of age) had OA. Considering this prevalence, one can estimate that presently, the total number of Brazilians with OA is almost 40 million (Figure 1).

Other epidemiological data are shown in Table 1.

**Table 1:** Epidemiological data (%) for OA in Brazil.

Gender	male=40.2 female=59.8
Age	63.7 (>60 years) 26.8 (41-60 years) 9.5 (<40 years)
Causes	36.0 (genetics) 20.0 (biomechanics) 16.7 (posttraumatic) 15.6 (inflammatory) 11.8 (unidentified)
BMI	46.0 (>30.0) 31.7 (25.0-29.9) 14.6 (18.5-24.9) 7.8 (<18.5)
Physical activity	48.3 (sedentary) 26.2 (professional practice) 25.5 (nonprofessional practice)
Affected joints	45 (knees) 31.4 (hands) 18.6 (hip) 17.4 (others)
Risk of diseases	29.0 (cardiovascular) 27.1 (gastrointestinal) 19.3 (renal)

In the population evaluated, the gender distribution of Brazilians with OA is 40.2% men and 59.8% women, indicating a proportion of 1.5 women to each man.

As for the age range, the distribution was 63.7%, 26.8% and 9.5% in the age groups >60 years, 41-60 years and <40 years, respectively.

Cases of OA with a pathogenesis linked to biomechanics represented little more than 1/3 of all patients. The cases arising from genetics constituted the second largest group, representing approximately 1/5 of the patients.

In relation to the distribution of patients according to body mass index (BMI), 46% of the patients were obese (BMI >30.0). Those who were overweight (BMI 25.0-29.9) represented just over 30% of the OA patients. In summary, the portion of patients above normal weight represented more than 3/4 of all patients.

According to the Delphi panel, almost half of the patients are sedentary. The regular practice of nonprofessional physical activity occurs in approximately 1/4 of the patients. A very similar result was found regarding the practice of professional physical activity.

### Clinical profile of patients according to the joints affected

Considering all individuals with OA, 45% had knee joint involvement. Cases involving the hand joints comprised the second largest group, while those affecting the hip represented less than 20% of patients.

The sum of the percentages reached 112.4%, signaling that just over 12% of the patients had more than one joint compromised.

### Clinical profile of patients by the presence of renal, gastrointestinal and cardiovascular risks

The presence of increased renal risk was found in approximately 1/5 of patients, while increased cardiovascular risk was found in 29% of patients. Increased gastrointestinal risk occurred in 27% of patients.

### Forms of treatment

The following are the findings for the forms of treatment undertaken by different specialists in OA of the knee, as determined by the SAC. The findings pertaining to the other affected joints are the subject of future research.

### No form of treatment

**Figure 2:**
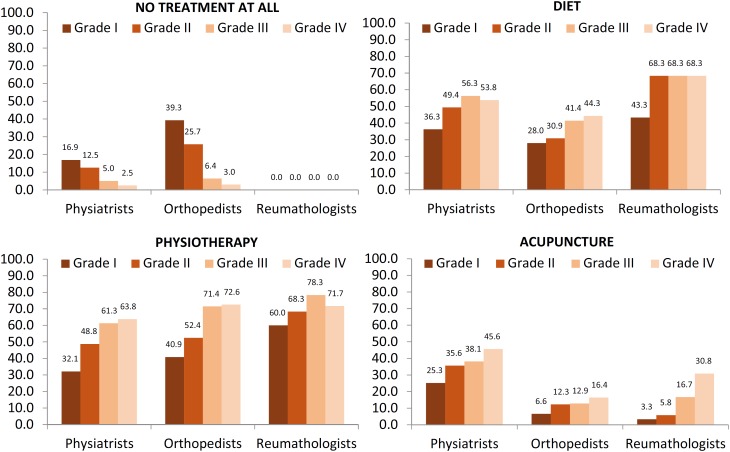
Nonpharmacological treatments of OA in Brazil among specialties

The findings related to “no form of knee OA treatment” reported by the Delphi panel varied largely, not only among the specialties but also according to the clinical condition of the patients. The rheumatologists established at least one form of treatment for patients with knee OA, regardless of the grade of radiographic Kellgren & Lawrence classification. Orthopedic surgeons are especially involved with treating patients who have degrees III and IV of OA. For physiatrists, intervention increases with the severity of the disease and was greater than the orthopedic approach and less than the rheumatological approach at all stages (Figure 2).

### Nonpharmacological treatment

For nonpharmacological treatment, a hypocaloric diet was recommended by all three types of specialists and was similar for varying degrees of involvement, especially for the most pronounced forms of the disease. Notably, the rheumatologists started the diet in more than 40% of patients at the earliest stage of the disease (Figure 2).

The same behavior pattern was found with the indications for physiotherapy (Figure 2).

The results still show that acupuncture is also a therapeutic modality indicated mainly by physiatrists for all degrees of the disease. This modality, however, is less adopted both by orthopedists and rheumatologists, with the latter using acupuncture in less than 30% of the patients with radiographic grade IV OA (Figure 2).

### Pharmacological treatment

#### Oral nonsteroidal anti-inflammatory drugs (NSAIDs)

**Figure 3:**
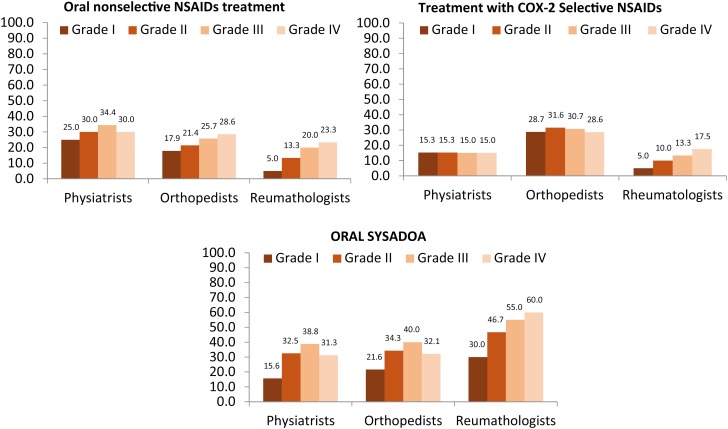
Pharmacological treatment among specialties

The prescription use of NSAIDs is seen in three specialties at all stages of knee OA, and in the more advanced stages of the disease (stages III and IV), approximately 54% of patients receive NSAIDs prescribed by all three types of specialists (Figure 3).

#### Oral pharmacological treatment with nonselective NSAIDs in fixed combination with a coater

The level of prescription of this class of treatments is lower than that of the isolated form of NSAIDs, except among rheumatologists who prescribed the fixed combination with the gastric protector for slightly more than 40% of the knee OA patients in the most severe OA class.

#### Oral pharmacological treatment with selective NSAIDs for COX-2

Orthopedic surgeons maintain higher levels (20%) of using these agents for their OA patients, especially in the more advanced degrees of the disease. Physiatrists and rheumatologists restrict the use of this class in patients with OA (Figure 3).

#### Oral pharmacological treatment with symptomatic slow-acting drugs for osteoarthritis (SYSADOA)

As for oral pharmacological treatment with SYSADOA, the rheumatologists prescribe them to approximately 30% of individuals with the initial forms of OA pathology and for half or more of the patients in the most advanced stages of the disease. Physiatrists and orthopedic surgeons prescribe in a very similar way at all stages, increasing progressively from the first to the third phase and decreasing in phase IV, when OA is more serious (Figure 3).

**Figure 4:**
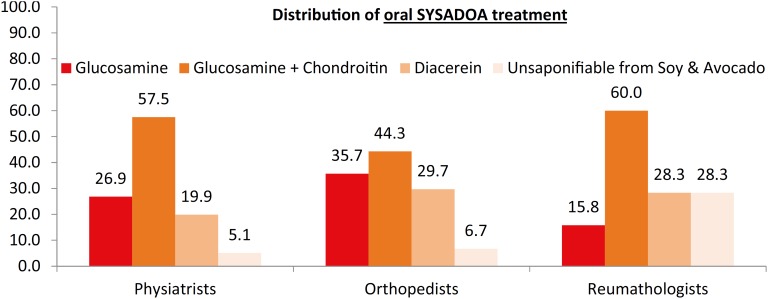
SYSADOA options for AO treatment among specialties

When the question was more directed, asking the specialists to opt for isolated glucosamine, glucosamine + chondroitin, diacerein or soy and avocado proteins, the most used among the 3 specialties was glucosamine + chondroitin, followed by isolated glucosamine for physiatrists and orthopedic surgeons (Figure 4).

#### Oral pharmacological treatment with serotonin reuptake inhibitors and/or norepinephrine

Regarding the prescription of serotonin reuptake inhibitors and/or norepinephrine, orthopedic surgeons are the ones who use this class of drugs the least. Physiatrists prescribe them more frequently for knee OA, while rheumatologists indicate this therapy for the most advanced forms of OA.

#### Oral pharmacological treatment with opioids

The rheumatologists use these agents with more than 40% of the patients with knee OA in the most severe phase of the disease. Physiatrists also prescribe them, followed by orthopedic surgeons.

#### Articular pharmacological treatment with corticosteroids

The greatest use of corticosteroids was by rheumatologists. The physiatrists are the experts who use them the least.

#### Articular pharmacological treatment with hyaluronic acid

The rheumatologists indicated hyaluronic acid treatment more frequently for grade III than for degree IV knee OA. Orthopedic surgeons indicated the application of intraarticular injections in approximately 30% of patients with degree III and in 22% of those with degree IV knee OA. The physiatrists use it for approximately 10% of patients with OA of knee grades II, III and IV.

#### Surgical treatment

The data show that most of the patients who are referred for surgical procedures by the three types of specialists involved are diagnosed with grade IV knee OA.

## DISCUSSION

This study traced a comprehensive study of the epidemiological profile of OA in Brazil, including prevalence information, risk factors and options for treatment of the disease, through the consensus opinion of 21 specialists in the areas of rheumatology, physiatry and orthopedics. We used the Delphi approach, which provides a transparent, explicit and extensive method for the closest possible identification of the current reality of OA in our country. The use of this technique is intended for situations characterized by the absence of data, lack of historical data, necessity of an interdisciplinary approach or for the generation of new ideas. The Delphi method also aims to predict future trends of the subject under study in the sense of extracting the structural perspectives.

The results presented herein reflect the consensual practice of specialists from different Brazilian regions in relation to treatment approaches for patients with symptomatic knee OA. The results thus provide robust evidence regarding the prevalence of OA and the treatment options.

We found that OA had a prevalence of 33% in the Brazilian adult population. The epidemiological studies available indicate that OA affects 10-15% of the world's population, presenting an incidence of more than 60% of men and 70% of women over 65 years of age (10). Regarding the prevalence, OA increases with age and is unusual in individuals under 40 but more frequent after the age of 60 (11). Our data match the worldwide findings. The precariousness of radiographic examinations for the early diagnosis of OA is also highlighted because radiography only presents changes after the disease has irreversibly compromised the tissues, especially cartilage. This fact recently led to the European League of Associations of Rheumatology (EULAR) to recommend that for clinical signs suggestive of OA, radiographic examination is not necessary to initiate treatment (12). Among joint diseases, OA is the most common, and it is estimated that one third of adult individuals between 25 and 74 years old present radiological evidence of OA in at least one joint (2). That study reinforces the findings found in the present research.

We also found a 1.5:1 ratio of women to men with OA. This result is in accordance with those of Lawrence et al. (2) who showed that the incidence of OA in the hand, hip, and knee increases with age, and women have higher incidence rates than men do, especially after the age of 50 years, possibly due to the hormonal imbalance that strikes women in the most advanced stages of adult life. Obesity is also a strong risk factor for the development of OA. Data show that OA is associated with metabolic syndrome, which implies a common pathogenic mechanism involving metabolic abnormalities and systemic inflammation (13–16). In studies using NHANES III data, there was a 5.26-fold increased risk of metabolic syndrome in individuals with OA at the age of 43.8 years (17–19). The present study found that 46% of the patients are obese, with a BMI above 30.0. Those who were overweight (BMI 25.0-29.9) represent a few more than 30% of the patients. Together, both groups represent 3/4 of all OA patients.

The objectives of OA treatment include pain relief and functional improvement (20).

In clinical practice, treatment should be based on individual evaluation, taking into account their needs and preferences, or the subjective interpretation of the evidence by the doctor (21). A gold standard treatment does not yet exist according to medical societies that most often deal with OA. Most likely due to the different phenotypes, OA in different joints is also treated different. Indications for knees are not the same as those for the spinal column or hand joints.

OA treatment can be divided into nonpharmacological, pharmacological and surgical interventions, the latest for the most severe cases. More than 90% of orthopedic surgeons report that they do not prescribe any treatment for Kellgren & Lawrence grades I and II OA. This result can be explained by the specialty profile because orthopedic surgeons are requested for more severe cases for surgical treatment. Alternatively, rheumatologists will initiate at least one type of treatment in all grades of OA.

Nonpharmacological measures include patient education, the use of heat and ice, weight loss, exercises, the use of physical support and occupational therapy and the removal of heaviness in joints such as the knees and hips (22).

Several of these interventions are part of physiotherapy treatment, which is divided among appropriated, uncertain and not appropriated treatment (23). This issue may be one reason why, in our query, when asked about the indication for physiotherapy as a global treatment, the physiatrists were less likely to prescribe physiotherapy at all stages.

For the treatment of knee OA, pharmacological recommendations from the American College of Rheumatology (ACR) include the use of acetaminophen, oral and topical NSAIDs, tramadol and articular injection of corticosteroids. The indication for surgery may be necessary when other treatments fail (22).

There are also the so-called “symptomatic slow-acting drugs in osteoarthritis” (SYSADOA), a description attributed to the fact that their effects, including the reduction in pain, require long-term administration to be noticeable (24). Among the drugs currently included in this category are chondroitin sulfate and chondroitin hydrochloride, glucosamine sulfate (24–26) and diacerein (27). When compared to NSAIDs, pain reduction and functional improvement are similar (24). However, considering tolerance and the reduced incidence of adverse effects and almost total absence of serious effects, SYSADOA have advantages over NSAIDs.

The last algorithm recommended for the management of knee OA elaborated by the European Study of Clinical and Economics Aspects of Osteoporosis and Osteoarthritis (ESCEO) (24–26,28,29) places “basic” pharmacological treatment using SYSADOA as the first step, more specifically the use of glucosamine with or without chondroitin. Our results showed that the combination of glucosamine and chondroitin is the most prescribed SYSADOA among the 3 categories, followed by isolated glucosamine, commonly prescribed by orthopedics and physiatrists.

The SAC drew attention to the fact that in general, clinical studies involve both chondroitin sulfate and chondroitin hydrochloride. This mixture of sulfate and hydrochloride can compromise clinical effects because sulfate is actually quite effective, unlike hydrochloride, whose results seem to contaminate the general findings (30).

The SAC data indicate that many patients, regardless of the site of OA, are largely not receiving any treatment, mainly those treated by orthopedists.

The SAC alerts to this fact and recommends that from the first clinical encounter, including the earliest stages of the disease, there be intervention as soon as the diagnosis is made because OA today is known as a disease that is liable to change. There are medicines capable of preventing and slowing the evolution of the disease. Prevention of the most advanced degrees of OA is of primary relevance. The inability to work and the reduction in quality of life greatly impacts the costs to the health and pension systems, whether in terms of treatment or in early retirement from functional disability (31).

The Delphi panel was instructed to report the current medical treatment in their respective regions, including the standards adopted for the treatment of patients with OA and not only individual experience. The panel relied on participants who were appointed by the scientific advisory committee, which was responsible for coordinating this research. Thus, it provided the best available evidence regarding the use of pharmacological and nonpharmacological options available for treatment. In addition, the Delphi panel received formal instruction on the application of the methodology. Therefore, the results presented herein reflect the consensual practice of the specialists in the treatment approaches for patients with symptomatic OA. These results are expected to be useful for the development of quality of life measures for OA patients. We also understand that new evidence is still being developed, which will be disseminated in future updates.

## CONCLUSION

Osteoarthritis (OA) is a common form of joint disease, affecting 33% of the Brazilian adult population. There is a large percentage of patients who do not receive any form of treatment in the earliest stages of the disease. The glucosamine + chondroitin combination is the most prescribed treatment by all three types of specialists, mainly in the initial phases of OA. The experience and different approaches of different types of specialists change the way of treating OA patients.
